# Molecular Mirror Technology Facilitates High-Throughput, Accurate SARS-CoV-2 Testing

**DOI:** 10.1128/spectrum.00392-21

**Published:** 2021-08-25

**Authors:** Susan Realegeno, Sara Hash, Charlene Wong, Roland Liu, Jovan Shepherd, Robert T. Schooley, David A. Lipson, Frederick Fung, Suresh Menon, David T. Pride

**Affiliations:** a Department of Pathology, University of California, San Diego, La Jolla, California, USA; b Menon Biosensors, Inc., Escondido, California, USA; c Department of Medicine, University of California, San Diego, La Jolla, California, USA; d Department of Biology, San Diego State University, San Diego, California, USA; Memorial Sloan Kettering Cancer Center

**Keywords:** COVID-19, Molecular Mirror, SARS-CoV-2, automation, nuclear magnetic resonance, nucleic acid amplification

## Abstract

Tests to diagnose acute SARS-CoV-2 infection are at the center of controlling the COVID-19 pandemic. Rapid tests benefit from providing quick results but suffer from lower sensitivity, while PCR tests usually take longer to provide more reliable results and can be difficult to scale to meet population needs. We evaluated the diagnostic efficacy of a Molecular Mirror assay (MMA) using nucleic acid extraction and a nucleic acid extraction-free method to determine its ability to identify SARS-CoV-2 in nasal specimens from individuals suspected of having SARS-CoV-2. We compared the MMA using nucleic acid extraction to the emergency use authorization (EUA)-approved TaqPath reverse transcriptase PCR (RT-PCR) assay to determine its performance characteristics. From 412 total specimens (including 115 previous positives and 297 previous negatives), we found that the positive percent agreement (PPA) was 99.1% (confidence interval [CI], 97.4% to 100.0%) and the negative percent agreement (NPA) was 99.3% (95% CI, 98.4% to 100.0%) for SARS-CoV-2 detection. Using the extraction-free method, we analyzed 109 specimens (51 previous positives and 58 previous negatives) and found that the PPA for the more rapid version of the assay was 87.8% (95% CI, 78.5% to 96.9%) and the NPA was 100.0% (95% CI, 100.0%) for virus detection. The extraction method has performance comparable to what is observed in many PCR-based assays. The extraction-free method has lower PPA but has the advantage of being more rapid and having a higher throughput. Our data offer a proof of concept that nuclear magnetic resonance (NMR) detection can be used in SARS-CoV-2 diagnostic testing and may allow for alternative supply chains to increase testing options.

**IMPORTANCE** Accurate diagnostics for SARS-CoV-2 infections have been critical for responding to the COVID-19 pandemic. Both high-sensitivity/specificity PCR-based tests and lower-sensitivity/specificity rapid antigen assays have been the subject of worldwide supply chain limitations as individual facilities and countries have struggled to meet their population testing needs. We evaluated the diagnostic efficacy of a Molecular Mirror assay (MMA), which uses nuclear magnetic resonance to detect the presence of SARS-CoV-2 nucleic acids both with and without full nucleic acid extractions. We found that compared to a U.S. emergency use authorization (EUA) approved assay (TaqPath) that uses reverse transcriptase PCR (RT-PCR), the MMA had high PPA and NPA with full nucleic acid extractions, and acceptable positive percent agreement (PPA) and negative percent agreement (NPA) with an extraction-free protocol. In a landscape marred by supply chain shortages across the world, altered SARS-CoV-2 detection methods such as the MMA can add to testing supplies while providing quality SARS-CoV-2 testing results.

## INTRODUCTION

Severe acute respiratory syndrome coronavirus 2 (SARS-CoV-2) was first identified in December 2019 and is the causative agent of a global pandemic. Infection with SARS-CoV-2 can manifest as a range of symptoms from asymptomatic to a severe pulmonary disease, known as SARS-CoV-2-associated coronavirus disease 2019 (COVID-19) (1). Since transmission can occur in both asymptomatic and symptomatic individuals (2–4), laboratory testing is particularly important in the diagnosis of COVID-19 to prevent further spread of the virus.

The urgent demand for diagnostic testing has challenged many clinical laboratories to provide access to high-volume testing (5). Widespread supply shortages and availability of commercial assays has made it difficult to meet the worldwide demand for diagnostic testing (6). The current standard of care diagnostic methods for SARS-CoV-2 are nucleic acid amplification tests (NAATs), such as real-time reverse transcriptase PCR (RT-PCR). Many of these have received emergency use authorization (EUA) in the United States from the Food and Drug Administration (FDA) and have been used in both symptomatic and asymptomatic individuals to help identify those who can potentially transmit the virus. Some have instituted protocols that include periodic SARS-CoV-2 testing to identify those infected and remove them from the population to prevent ongoing virus transmission. Several institutions have used such programs to reopen their in-person learning programs (7–10).

Because of the relative lack of available consumables and demand for high-volume testing for SARS-CoV-2, there have been efforts by other groups to explore alternative testing approaches. For example, there is a myriad of rapid antigen tests by the FDA that detect viral proteins authorized. While some of these tests are now readily available, emerging data have shown that they lack sensitivity compared to NAATs for use in the broader population (7, 11–13). Other groups have developed rapid molecular assays using loop-mediated isothermal amplification (LAMP) (14) or CRISPER-Cas12 technology (15, 16), while some have focused on high-throughput approaches such as sequencing (17, 18).

In order to meet the demand for more testing, high-throughput methods are critical, and preferably methods that can take advantage of easy-to-obtain consumables and preexisting equipment already in place across the country. We explored nuclear magnetic resonance (NMR) as an alternative modality for the detection of SARS-CoV-2 RNA in clinical samples. NMR-based technology has been previously shown to successfully detect pathogens in clinical specimens. For example, this approach has been used to detect Clostridium difficile in stool specimens with acceptable performance characteristics (19) as well as Vibrio parahaemolyticus and Salmonella spp. in food samples (20, 21). Now, this study demonstrates that NMR-based technology can also be used for the detection of SARS CoV-2 in clinical samples using either nucleic acid extractions or extraction-free methods. The benefit of NMR is that it can be adapted to much higher throughput technology such as magnetic resonance imaging (MRI). Here, we evaluate a molecular-based method that uses NMR technology for the detection of SARS-CoV-2 nucleic acid in clinical respiratory specimens. Our goals were to demonstrate the potential for NMR to detect SARS-CoV-2 compared to a PCR-based assay, to determine its analytical performance, and to provide a framework by which this technology may be spread across the world to further SARS-CoV-2 detection.

## RESULTS

### Study population for nucleic acid extraction MMA protocol.

We examined 412 previously collected specimens in our validation of the Molecular Mirror assay (MMA). The population included individuals aged from 1 year old to 84 years old, with an average age of 43.09 ± 18.49 years. It included 193 males (46.84%) and 219 females (53.16%). All samples were tested on the Thermo Fisher TaqPath SARS-CoV-2 EUA assay in the course of routine standard-of-care at UC San Diego Health. The TaqPath assay detects three different SARS-CoV-2 genes, the ORF1ab gene, S gene, and N gene. Identifying 2 of the 3 targets in each clinical specimen is sufficient to make the diagnosis of SARS-CoV-2 infection. Of the 412 specimens tested, 115 were positive for the SARS-CoV-2 virus using the TaqPath assay. The cycle threshold (*C_T_*) values for all genes generally ranged from 8 to 40, with the S gene not detectable in only a single specimen; however, in that same specimen, the ORF1ab and N genes were detected.

### Probe analysis.

To help decipher potential specificity for the probes used in the MMA, probes 1 and 2 for the MMA were subjected to a BLAST search against working sets of SARS-CoV-2 genomes (*n* = 11,629) and non-SARS-CoV-2 genomes (*n* = 585), which include the SARS-CoV-1, Middle East respiratory syndrome (MERS), HKU1, 229E, OC43, and NL63 strains. Probe 1 (Table 1) matched exactly to 98.72% of the SARS-CoV-2 working set and closely matched (1 to 2 mismatched bases) 1.24%. The strongest matches in the non-SARS-CoV-2 working set (56.41%) to probe 1 had a bitscore (measures sequence similarity independent of database size) of 20, corresponding to only a 10-bp sequence match within the 20-bp probe. Probe 2 matched exactly to 99.66% of the SARS-CoV-2 working set and closely matched (1 to 2 mismatched bases) the remaining 0.34%. The strongest matches in the non-SARS-CoV2 working set (8.89%) to probe 2 had a bitscore of 32, corresponding to 22/24 matched bases. However, the majority (90.77%) of the non-SARS-CoV-2 working set had a bitscore of 22 or lower, corresponding to 11-bp sequence or shorter matched to the 24-bp probe. We also evaluated the probes against several of the novel SARS-CoV-2 variants, including B.1.1.7, B.1.351, B.1.427/429, P.1, and B.1.525. Each of the probes was an exact match for each of these variants (see Tables S1 and S2 in the supplemental material).

**TABLE 1:** Probe specificity

Probe	SARS-CoV-2 genomes (%) (*n* = 11629)	Non-SARS-CoV-2 genomes (%) (*n* = 585)
Probe 1 Bitscore (Max 40)		
40	98.72	0.00
32–38	1.24	0.00
22–30	0.03	0.00
20	0.00	56.41
18	0.00	34.53
<18	0.01	9.06
Probe 2 Bitscore (Max 48)		
48	99.66	0.00
34–46	0.34	0.00
32	0.00	8.89
24–30	0.00	0.34
22	0.00	5.81
20	0.00	53.50
18	0.00	31.45
<18	0.00	0.00

### *C_T_* value ranges for nucleic acid extraction MMA protocol.

We did not choose the SARS-CoV-2-positive specimens for use in this study based on prior *C_T_* values. However, we did evaluate the *C_T_* values for the ORF1ab, N, and S genes among the 115 previously positive specimens. We focused on N gene *C_T_* values to compare to MMA since this gene is a shared target between the two assays. We found that 36 of the specimens had *C_T_* values of ≤15.00, 37 ranged from 15 to 20, 17 from 20 to 25, 22 from 25 to 30, and 3 specimens had *C_T_* values of >30. This range of positive values allowed us to test the MMA across a variety of virus relative abundances.

### Clinical performance of nucleic acid extraction MMA protocol for SARS-CoV-2 detection.

**FIG 1:**
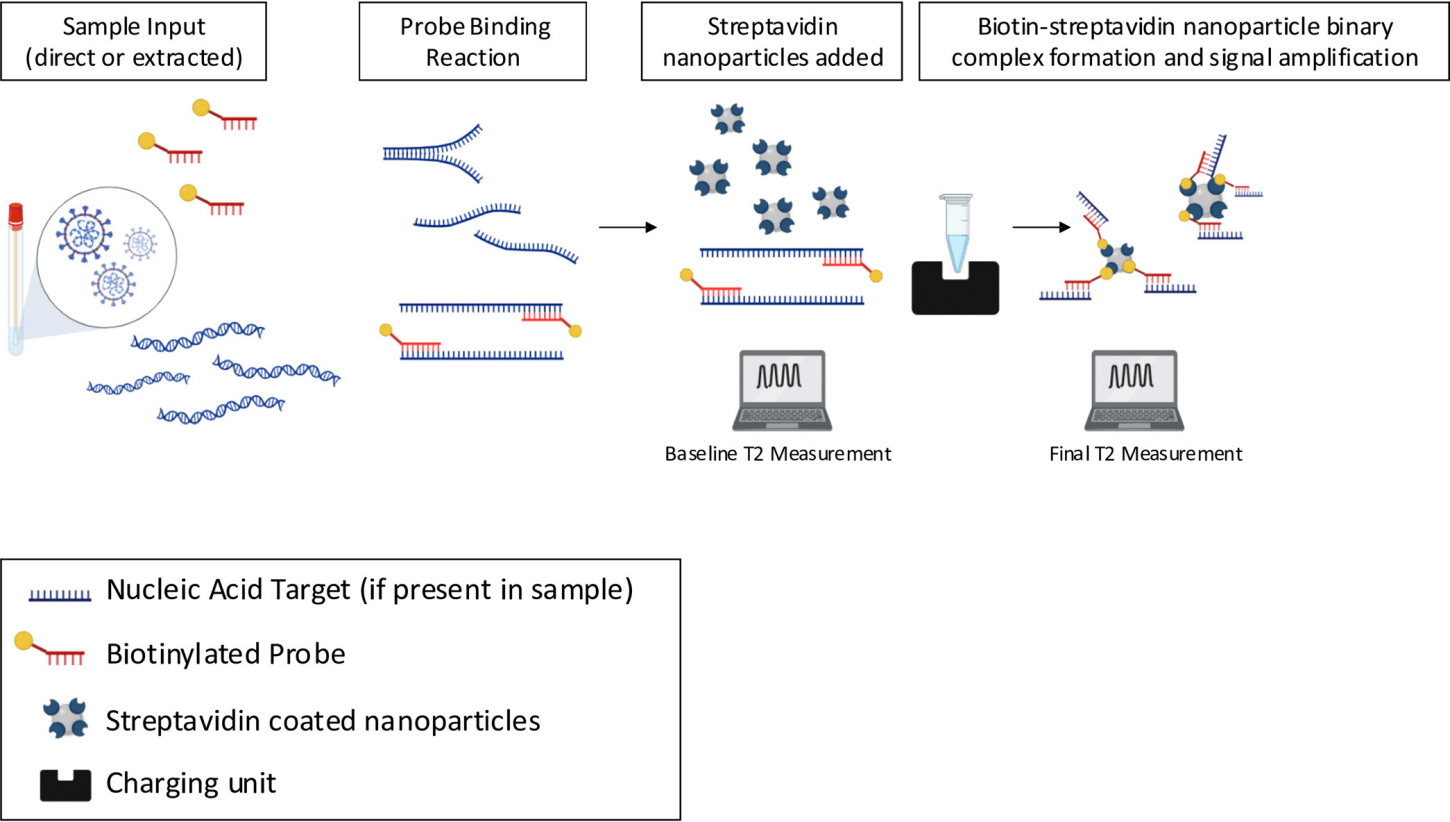
Diagram of the Molecular Mirror assay workflow.

We developed the MMA to be scalable for processing large numbers of SARS-CoV-2 tests. The protocol involves collection of swabs from individuals, performing RNA extraction on those swabs, adding SARS-CoV-2-specific probes to the resulting RNA, allowing for probe binding to occur during a thermocycling step, clustering with streptavidin nanoparticles, and detection of the bound probe/streptavidin clusters using nuclear magnetic resonance (NMR) (Fig. 1; Table S3). In an aqueous medium, which generally has a long T2 (transverse relaxation time; on the order of 2,000 msec), the addition of nanoparticles generally reduces the magnetic resonance property T2 when the nanoparticles are uniformly dispersed or suspended throughout the solution (22). This effect results from depolarization of adjacent water molecules, with each nanoparticle acting as a “depolarizing center”. However, if the nanoparticles in the solution become clustered through agglomeration, the number of depolarizing centers is reduced, and therefore, the effect on T2 is also reduced, with the result that the T2 of the aqueous solution is increased relative to the T2 of the medium when the nanoparticles are dispersed. Thus, when there is no probe bound, streptavidin/probe clusters do not form, and there is no change in T2 signal.

We examined the 412 total specimens using MMA, which included 115 previously positive and 297 previously negative specimens on the TaqPath assay. Of those 115 previously positive specimens, 114 tested positive on the MMA (Table 2). This equated to a PPA of 99.13% (95% confidence interval [CI], 97.4% to 100.00%) compared to the TaqPath result. The lone specimen that tested negative was the only specimen in which the N and ORF1ab genes were detected but the S gene was not detected. This specimen had ORF1ab and N gene *C_T_* values of 36.4 and 34.1, respectively, suggesting that it was close to the limit of detection for the TaqPath assay and at or above the limit of detection for the MMA. Of the 297 previously negative specimens, 295 tested negative on the MMA, which equated to an NPA of 99.33% (95% CI, 98.40% to 100.00%). Overall, 409 of the 412 total specimens tested provided the expected results, corresponding to an overall percent agreement (OPA) of 99.27% (95% CI, 97.00% to 100.00%). All specimens when tested were arranged in a checkerboard pattern during their processing, and no evidence of contamination events was identified (data not shown).

**TABLE 2:** PPA and NPA of MMA with nucleic acid extraction compared to TaqPath

Result type	NMR positive	NMR negative	Total
TaqPath positive	114	1	115
TaqPath negative	2	295	297
	**Value (%)**	**95% CI (%)**	
OPA	99.3	97.0 to 100.0	
PPA	99.1	97.4 to 100.0	
NPA	99.3	98.3 to 100.0	

To determine whether the results we obtained in our analysis of the MMA were reproducible, we also performed precision studies by evaluating 12 total specimens (3 previously negative and 9 previously positive specimens) over 3 consecutive days. Including a total of 5 replicates across the 3 days for each sample (60 replicates total), 59/60 (98.33%) of those provided the expected results. There was one previously positive specimen that tested positive in 4 of the 5 replicates; however, the value fell slightly below the limit of detection of the assay for one of the replicate samples.

**FIG 2:**
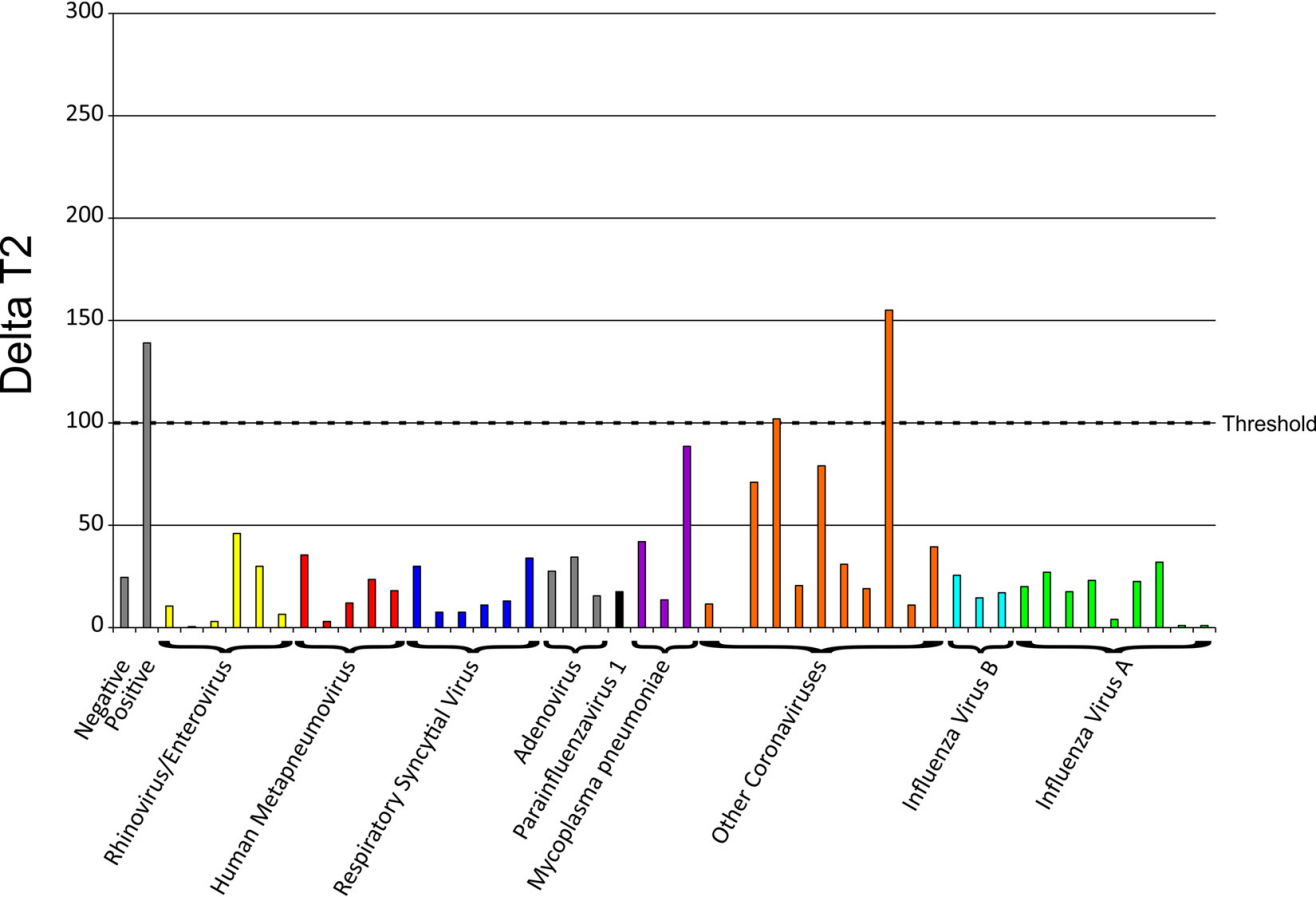
Bar chart representing delta T2 scores of the Molecular Mirror assay for 47 respiratory specimens that tested positive for viruses other than SARS-CoV-2. The *y* axis shows the viruses that each specimen tested positive for, and the *x* axis shows the delta T2 scores. The threshold cutoff for the Molecular Mirror assay is shown by the dotted line.

In our specificity analysis, we tested 250 previously negative SARS-CoV-2 specimens using the MMA and an additional 47 specimens that were previously positive for other known viruses to ensure that the MMA did not detect other common respiratory pathogens, including the seasonal coronavirus. The 47 specimens were first tested using the TaqPath assay to ensure that SARS-CoV-2 was not detected; all 47 were negative. These 47 specimens were then tested using the MMA. Of these 47 specimens, 6 were previously positive for rhinovirus/enterovirus, 5 for human metapneumovirus, 6 for respiratory syncytial virus, 3 for adenovirus, 1 for parainfluenza 1, 3 for Mycoplasma pneumoniae, 11 for coronaviruses (including HKU1, OC43, 229E, and NL63), 3 for influenza B, and 8 for influenza A. Of the 47 specimens, 45 provided the expected negative SARS-CoV-2 result (Fig. 2). Two specimens provided unexpected results, positive using the MMA, and were both previously positive for coronaviruses (HKU1, OC43, 229E, or NL63). Of note, both specimens had an MMA result at or near the cutoff for this assay. Probe specificity using the NCBI primer-BLAST tool resulted in SARS-CoV-2 sequences as the sole hit; no other organisms (bacterium, virus, or homo sapiens) shared a target sequence. Overall, the specificity of the assay was still 99.33% despite these discrepancies.

### Analytical sensitivity of the MMA using the nucleic acid extraction protocol.

**FIG 3:**
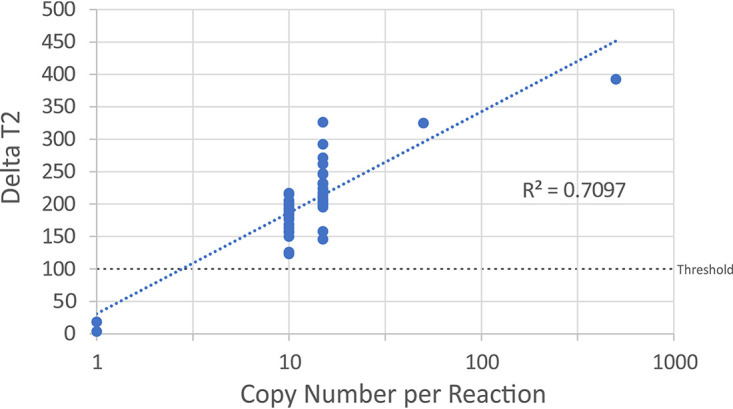
Scatterplot of delta T2 scores compared to the copy number of viruses used in the limit of detection testing for the nucleic acid extraction MMA protocol. The copy number of viruses per reaction is shown on the *x* axis, and the delta T2 score is shown on the *y* axis. The threshold for positivity is also shown by the black dashed line along the *x* axis. A linear regression line is drawn through the points and is represented by the dashed blue line. The *R*-squared value is shown above the threshold line.

We also examined the analytical sensitivity of the MMA to determine its relative ability to detect specific concentrations of the SARS-CoV-2 virus in contrived specimens ranging from 5 to 500 copies per reaction (1,000 to 100,000 copies/ml). A total of 20 replicates of contrived specimens each at 10 and 15 copies per reaction (2,000 and 3,000 copies/ml, respectively) were tested. All of the specimens were detected at 10 and 15 copies per reaction, indicating that the limit of detection for this assay is 10 copies of the SARS-CoV-2 virus per reaction. There was a linear relationship (*R*^2^ = 0.7097) between the copy number tested per reaction and the delta T2 value obtained for the MMA assay (Fig. 3).

### Extraction-free MMA clinical performance.

Because the MMA requires a full nucleic acid extraction, which results in the need for consumables that have been relatively constrained by supply chains (6), we evaluated the MMA without the nucleic acid extraction step to decipher how it compared with the TaqPath assay results. To perform the MMA without nucleic acid extraction, we first treated the samples with proteinase K, followed by inactivation at 95°C. From there, samples were treated exactly the same as is described in the MMA protocol by proceeding with the probe binding steps. The removal of the extraction step eliminated additional reagents and approximately 30 min to an hour from the protocol.

In our clinical evaluation of this protocol, we tested 109 total specimens (51 previously positive specimens and 58 previous negatives). Of these specimens, there was an equal age distribution (43.8 ± 19.0 for previous TaqPath positives compared to 45.4 ± 20.8 for previous negatives) and sex (56.9% female for previous positives compared to 58.8% female for previous negatives). Of those 51 previous TaqPath positives, 43 tested positive with the extraction-free method, while all 58 of the previous TaqPath negatives tested negative (Table 3; Table S4). These data correspond to a PPA of 87.8% (95% CI, 78.6% to 96.9%), an NPA of 100%, and an OPA of 94.4% (95% CI, 81.1% to 100.0%). While the PPA was lower than that for the full extraction method, it was superior to the performance of other available rapid assays (7, 12, 23).

**TABLE 3:** Sensitivity and specificity of MMA without nucleic acid extraction compared to TaqPath

Result type	NMR positive	NMR negative	Total
TaqPath positive	43	8	51
TaqPath negative	0	58	58
			
	**Value (%)**	**95% CI (%)**	
OPA	94.4	81.1 to 100.0	
PPA	87.8	78.7 to 96.9	
NPA	100.0	NA^*a*^	

a NA, no applicable confidence interval could be determined.

We noted that there was diminished PPA for the extraction-free MMA, so we evaluated whether there were specific *C_T_* value ranges for the TaqPath assay that did not test positive when evaluated with the MMA. We found that there was a correlation between *C_T_* values and the ability to detect SARS-CoV-2 in each sample, with specimens that had ORF1ab *C_T_* values of 27.5 or greater being less likely to be detected by the extraction-free MMA (Table S4). All specimens in which the ORF1ab *C_T_* value was >31 tested negative on this version of the assay.

Because of the differences in detection for the MMA and the extraction-free MMA, we also evaluated the limit of detection (LoD) of the extraction-free MMA to decipher whether the removal of the extraction step resulted in lower analytical sensitivity. As we previously detailed, the LoD of the extraction-based MMA protocol was 10 copies per reaction (2,000 copies/ml). In our analysis of the extraction-free MMA, we found that 50 copies per reaction (10,000 copies/ml) could be reliably detected, verifying that the removal of the extraction step resulted in an altered LoD for this assay (Fig. S1).

## DISCUSSION

The SARS-CoV-2 pandemic caught much of the world by surprise, with many countries unprepared to meet the demands for testing. More timely testing would have allowed for more effective isolation and quarantining to reduce viral spread. In the United States, over the course of the pandemic, it has been estimated that to significantly reduce virus spread, many more tests per day needed to be performed (8, 9). Thus, there is a significant need to identify alternative means to test larger numbers of people, with clinically useful results available in a relatively short time frame.

One of the biggest limitations with SARS-CoV-2 testing has been the availability of testing materials (6, 24). Many laboratories have been constrained by staffing problems, equipment availability, and supply chain constraints from various testing manufacturers, limiting the numbers of tests that they can perform. Numerous tests, including PCR-based assays and rapid antigen assays, entered the U.S. market to address supply chain constraints. These tests substantially improved the atmosphere for testing but came with a set of problems, many related to assay performance. While most PCR-based assays have both high sensitivity and high specificity, some rapid antigen tests have lower sensitivity and overall performance than can be tolerated in some applications. Indeed, recent studies have elucidated that rapid antigen-based assays have relatively poor overall predictive values for disease and may not be sufficiently rigorous to make isolation and quarantine decisions (11, 12). The significant benefit of the MMA is that it is molecular-based and holds promise to generate results that are highly sensitive and specific. We were only able to evaluate three positive specimens that had *C_T_* values ≥30, and one of those specimens tested negative. This suggests that the MMA could be less reliable for specimens with lower viral loads. One of the drawbacks of the full-extraction MMA method is that it requires many of the same materials that are also required for PCR; thus, it may not improve supply chain restraints. The extraction-free method relieves this requirement, but the PPA is reduced as a result. While there is generally lower PPA for this protocol, it is comparable to the sensitivities of other RT-PCR assays using extraction-free protocols (25, 26).

We evaluated the MMA in comparison to the Thermo Fisher TaqPath assay primarily because these assays can use shared equipment. For example, our laboratory uses Hamilton liquid handler instruments for specimen processing and reagent preparation, the KingFisher Flex for nucleic acid extraction, and the ABI 7500 Fast Dx instrument for thermal cycling and RT-PCR. The extraction-free version eliminated the need for the nucleic acid extraction. The same instruments can be used for MMA, but at the final detection step, it uses NMR instead. The MMA can be performed on other instruments aside from the ones described here and can be scaled to accommodate single tests or 96- or 384-well plates, but for maximum usability requires an NMR that also can read in a plate format. The extraction-free MMA method can be performed rapidly when testing single specimens, but when performed in multiwell formats, the time to result approaches those of extraction-free PCR.

Since the beginning of this pandemic, scientists have been searching for SARS-CoV-2 tests that are simple to perform and have performance characteristics similar to those of RT-PCR. The highlight of the MMA is that its performance was similar to that of traditional PCR with high sensitivities and specificities that resemble those found with other PCR assays for the MMA with nucleic acid extractions, but also with relatively high sensitivities and specificities when the nucleic acid extraction step is removed. While this evaluation of the MMA was specific for NMR, the same principles apply that could render it applicable to MRI. The NMR method we performed uses permanent magnets assembled to provide between 0.1 and 1.0 Tesla field strength, which essentially mimics what can be observed in MRI machines providing 1.0 to 5.0 Tesla. The NMR machine only took seconds to get each result. There are automated NMR instruments capable of significantly increasing the throughput of this assay.

Our specificity data suggest that the probes targeting the N gene are relatively specific for SARS-CoV-2 (Table 1) compared to other human coronaviruses. However, the cross-reactivity we observed against another coronavirus specimen indicates that there could be some cross-reaction from other coronaviruses. Such a phenomenon could potentially occur from consecutive bases that match over shorter portions of the probe sequence. Any potential specificity could be overcome by choosing primers for different SARS-CoV-2 genes or gene segments, increasing stringency of the binding solution, or altering probe binding temperatures.

### Conclusions.

There are a number of rapid antigen and more accurate PCR-based tests available for acute diagnostics of SARS-CoV-2 infections. What each of these test types have in common is that they do not offer the ability detect the probe-bound virus in mere seconds. This assay, particularly without the nucleic acid extractions, offers reasonable PPA and NPA, while eliminating some of the consumables that have been under widespread supply chain constraints. While tweaks to the technology to improve coronavirus specificity and to improve the extraction-free sensitivity are still ongoing, this technology holds the potential to improve throughput for SARS-CoV-2 detection.

## MATERIALS AND METHODS

### Clinical samples.

A total of 412 clinical respiratory samples collected were included in the study examining the MMA using nucleic acid extraction. These specimens consisted of 359 nasal, 52 nasopharyngeal, and 1 oropharyngeal specimen collected in viral transport media (VTM). All samples were processed at the UC San Diego Health Clinical Microbiology Laboratory for routine diagnostic testing using the TaqPath COVID-19 RT PCR assay. Of the total, 365 out of the 412 patient specimens were collected from November to December 2020 and processed within 24 h of collection for SARS-CoV-2 testing. The additional 47 specimens were collected from February to March of 2020 for respiratory pathogen detection other than SARS-CoV-2 testing and were previously positive for other pathogens such as influenza virus, coronavirus (229E, HKU1, NL63, and OC43), human metapneumovirus, respiratory syncytial virus, adenovirus, parainfluenza 1 virus, and Mycoplasma pneumoniae. Respiratory pathogen identification was performed on these 47 specimens using the respiratory pathogen panel on the ePlex system (GenMark Diagnostics, Carlsbad, CA). After routine testing, these sample were frozen at −80°C and subsequently thawed for retrospective SARS-CoV-2 testing using the TaqPath COVID-19 RT-PCR assay.

We also included an additional 109 specimens for the MMA extraction-free protocol collected from February to March 2021 in VTM. All 109 of those specimens had previously been tested using the TaqPath assay before testing with MMA, which included 51 previously positive specimens and 58 previously negative nasal specimens.

### TaqPath COVID-19 RT-PCR assay.

The TaqPath COVID-19 combo kit (Thermo Fisher Scientific, Waltham, MA) was used for the detection of SARS-CoV-2 RNA in clinical samples according to the manufacturer’s instructions outlined in the EUA. Sample and assay preparation was done using the Hamilton Star, an automated liquid handler (Hamilton Company, Reno, NV). Nucleic acid (NA) extraction was performed using the MagMax viral/pathogen nucleic acid isolation kit and the KingFisher Flex purification system (Thermo Fisher Scientific). RNA extraction from specimens was then used as a template for the real-time RT-PCR, which targets the SARS-CoV-2 S (spike), N (nucleocapsid), and ORF1ab (polyprotein) genes, performed on the Applied Biosystems 7500 Fast DX real-time PCR instrument (Thermo Fisher Scientific). RT-PCR data were analyzed using the COVID-19 Interpretive Software v2.5, and qualitative results were provided by the software. Positive results were based on detection of at least 2 of the 3 SARS-CoV-2 targets. Cycle threshold (*C_T_*) values for each gene were also obtained using the interpretive software for each sample identified as positive.

### Molecular Mirror SARS-CoV-2 assay.

Direct clinical specimens as well as extracted nucleic acid from clinical specimens served as the input for the Molecular Mirror assay. For the direct specimen input, clinical samples were first treated with proteinase K at 2.5 mg/ml (Thermo Fisher Scientific, Waltham, MA) at room temperature (approximately 22°C) for 1 min and then heat inactivated at 95°C for 5 min before proceeding to the probe binding step (see below). In addition, extracted nucleic acid was obtained from routine SARS-CoV-2 testing using the TaqPath COVID-19 RT-PCR protocol. To evaluate for cross contamination, when possible, positive and negative samples were arranged in a checkerboard fashion and tested by a separate operator in a blinded fashion.

In brief, the Molecular Mirror technology is a novel and patented approach that uses biotinylated probes capable of binding to nucleic acid of the target microorganism (U.S. Patent no. 9,442,110 B2). High-pressure liquid chromatography (HPLC)-purified lyophilized biotinylated probes (IDT, Coralville, IA) were resuspended in 10 mM Tris/1 mM EDTA buffer. These probes target the SARS-CoV-2 N gene and were added to the direct patient sample or extracted nucleic acid, followed by a probe binding reaction to target nucleic acid in the test samples (nCoV_N1 [probe 1]: 5′-GAC CCC AAA ATC AGC GAA AT-3′ and nCoV_N2 [probe 2]: TCT GGT TAC TGC CAG TTG AAT CTG). The following cycling conditions were used: 25°C for 2 min, 53°C for 10 min, 95°C for 2 min, and 33 cycles of 95°C for 15 sec followed by 60°C for 1 min.

Streptavidin-coated nanoparticles were combined with the binding reaction in individual tubes at a final concentration (2 ng/μl), which allows for maximum surface coverage of the probes bound to the target viral nucleic acid. The baseline NMR spin-spin relaxation time (T2) signal was first measured (milliseconds) using the Lab-in-the-Box NMR system (Menon Biosensors, Inc., Escondido, CA), which is a measure of the T2 relaxation time before probe-bound target is reacted with the nanoparticles. To allow for the formation of a biotin-streptavidin nanoparticle binary complex and signal amplification, samples were placed in the Lab-in-the-Box charging unit for 10 min.

After the signal amplification incubation, a final T2 measurement was taken in duplicate and averaged. The delta T2 was calculated by subtracting the baseline signal from the average final T2.

A positive and a negative control were included in each batch of prepared samples. Extracted VTM and previously negative specimens served as negative controls, and contrived positive controls were prepared using 5,000 copies per ml of the nonreplicative recombinant SARS-CoV-2 virus (Accuplex SARS-CoV-2 reference material; SeraCare Life Sciences, Inc., Milford, MA) in verified negative patient specimens. The threshold value at which positive and negative results are differentiated was determined based against an analyte calibration curve using synthetic SARS-CoV-2 RNA (control 2, GenBank accession no. MN908947.3 Wuhan-Hu-1; Twist Bioscience, San Francisco, CA) prepared in extraction buffer and negative extraction buffer for comparison. A calculated delta T2 of 100 ms or greater was used as the threshold value at which a sample was identified as positive for SARS-CoV-2.

### Probe analysis.

Working sets of SARS-CoV-2 genomes and non-SARS-CoV-2 genomes were obtained using the NIAIN Virus Pathogen Database and Analysis Resource (ViPR) (27; http://www.viprbrc.org). All complete coronavirus genomes were filtered for having a human host and then parsed based on genome name and entry date. The resulting working sets consisted of 11,629 SARS-CoV-2 genomes (containing any of “SARS-CoV-2,” “HCoV-19,” or “severe acute respiratory syndrome coronavirus 2” and submitted on or after 2019) and 585 non-SARS-CoV-2 human coronavirus genomes (not containing any of the previous names and submitted before 2019). Probes 1 and 2 were subjected to BLAST searches against both sets using the inbuilt ViPR BLAST tool with an expected threshold of 1,000 and word size of 11 (SARS-CoV-2 set) or reduced to 7 (non-SARS-CoV-2 set) to allow for more inexact matches. Gap costs were 5 for existence and 2 for extension.

To evaluate probe matches to the variants, five genomes from each variant lineage (B.1.351, B.1.427/429, B.1.525, B1.1.17, and P.1) were selected for having originated from diverse geographical locations and were acquired from the GISAID database (gisaid.org) (28). Probes 1 and 2 were aligned to each genome and analyzed using the Geneious Prime v2021.0.3 Map to Reference tool.

### Limit of detection (LoD) and precision studies.

LoD studies for both the extracted and nonextracted protocol were performed using the full-genome Accuplex SARS-CoV-2 verification panel (SeraCare Life Sciences, Inc., Milford, MA) prepared in verified negative patient samples. Concentrations ranging from 5 to 500 copies/reaction (1,000 to 100,000 copies/ml) were evaluated in a final reaction volume of 5 μl, and 20 replicates were tested at the LoD. In addition, precision studies were performed using previously negative and previously positive clinical samples over 3 days, including running the specimens in triplicate on at least 1 of the 3 days.

### Data analysis.

Performance specifications were calculated using MedCalc v19.6.4 (MedCalc Software, Ostend, Belgium), including sensitivity, specificity, positive percent agreement, negative percent agreement, overall percent agreement, and accuracy. Also, 95% Clopper-Pearson confidence intervals were calculated for each parameter. Results from the TaqPath COVID-19 RT PCR assay served as the reference result. Regression analysis was performed using Microsoft Excel 365 (Redmond, WA). The workflow diagram was created using BioRender.
