# Supplementary material for: Molecular Mirror Technology Facilitates High-Throughput, Accurate SARS-CoV-2 Testing
Source: Microbiol Spectr. 2021 Aug 25;9(1):10.1128/spectrum.00392-21. doi: 10.1128/spectrum.00392-21 (PMC8552600; doi:10.1128/spectrum.00392-21)
Supplement: SUPPLEMENTAL FILE 1 — Supplemental material. Download SPECTRUM00392-21_Supp_1_seq5.pdf, PDF file, 0.2 MB [file spectrum00392-21_supp_1_seq5.pdf]

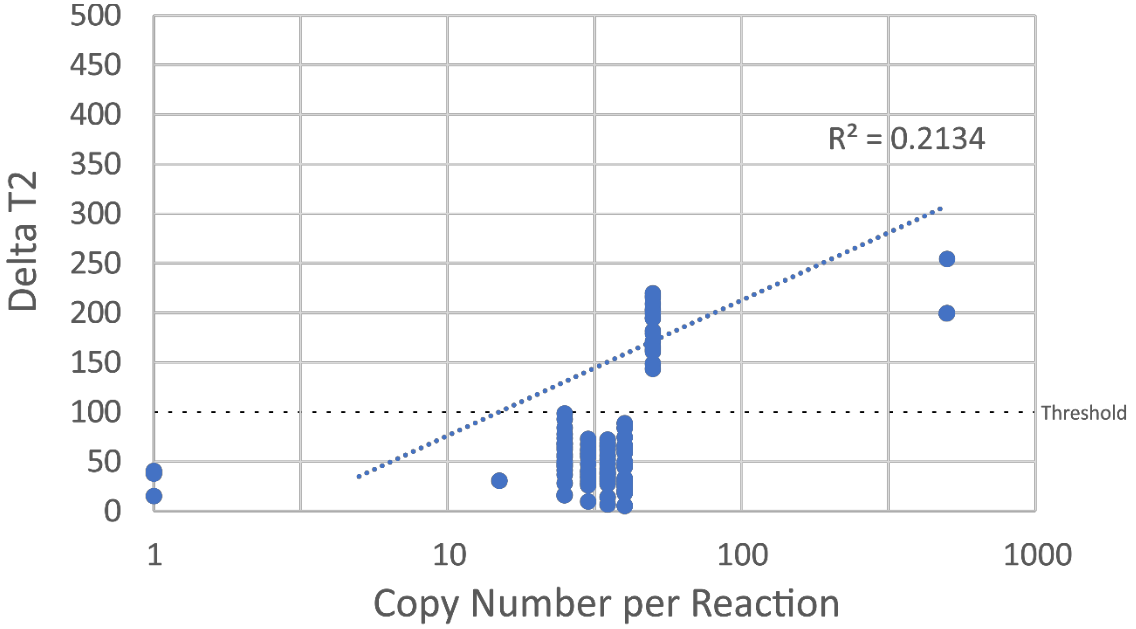

Figure S1: Scatter plot of Delta T2 scores compared to the copy number of virus used in the limit of detection testing for the nucleic acid extraction-free MMA protocol. The copy number of virus per reaction is shown on the x-axis, and the Delta T2 score is shown on the y-axis. The threshold for positivity is also shown by the black dashed line along the x-axis. A linear regression line is drawn through the points and is represented by the dashed blue line. The R-squared value is demonstrated above the threshold line.

Table S1: Probe specificity against variant genomes

Variant Lineage (n=5)	Average Probe 1 Identify	Average Probe 2 Identity
B.1.351	100%	100%
B.1.427/429	100%	100%
B.1.525	100%	100%
B.1.1.7	100%	100%
P.1	100%	100%

Table S1: Average percent identity for MMA probes against SARS-CoV-2 variants.
